# Mechanomyographic responses for the biceps brachii are associated with failure times during isometric force tasks

**DOI:** 10.14814/phy2.13590

**Published:** 2018-02-21

**Authors:** Joshua C. Carr, Travis W. Beck, Xin Ye, Nathan P. Wages

**Affiliations:** ^1^ Biophysics Laboratory Department of Health & Exercise Science University of Oklahoma Norman Oklahoma; ^2^ Neuromuscular Laboratory Department of Health, Exercise Science & Recreation Management University of Mississippi University Mississippi; ^3^ Ohio Musculoskeletal and Neurological Institute Department of Biomedical Sciences Ohio University Athens Ohio

## Abstract

In order to characterize the physiological adjustments within the neuromuscular system that contribute to task failure, this study examined the surface mechanomyographic (MMG) response during maximal and submaximal isometric force tasks of the elbow flexors sustained to failure. The time and frequency components of the MMG signal have shown to be influenced by motor unit activation patterns as well as tetanus. Therefore, it was hypothesized that the rate of change for the MMG response would associate with failure times and would be reduced to a similar degree between the two tasks. The isometric force tasks were performed by the dominant elbow flexors of twenty healthy males (age: 25 ± 4 years) and MMG was collected from the biceps brachii. Regression analyses were used to model the relationships between the rates of change for MMG versus failure times. There were high levels of interindividual variability in the response patterns, yet the models demonstrated significant negative associations between the rate of change for the MMG responses and failure times during both tasks (*R* ^2^ = 0.41–0.72, *P* < 0.05). Similarly, the mean MMG amplitude and frequency values were reduced to comparable levels at the failure point of the two tasks. The results of this study demonstrated that force failure is associated with the rate of diminution in the properties of the muscle force twitch.

## Introduction

The mechanomyographic (MMG) signal is composed of the low‐frequency lateral oscillations that resonant from the unfused, active muscle fibers within the frequency range of ~2–120 Hz (Barry and Cole 1990; Orizio 1993; Beck et al. 2008). The MMG signal is generated by dimensional changes in muscle fiber diameter that occur in response to motor unit activation (Frangioni et al. 1987; Orizio 1993). It is believed (Gordon and Holbourn 1948; Orizio et al. 2003; Beck et al. 2007a) the signal reflects the mechanical properties of muscle contraction and therefore contains information regarding motor control patterns (i.e., the relative contribution of motor unit recruitment and firing rate). The MMG signal has been used to examine motor unit function and the corresponding force twitch kinetics in the presence of fatigue (Yoshitake and Moritani 1999; Bichler and Celichowski 2001; Yoshitake et al. 2002). Though the signal is mechanical in nature, it has a direct, nonlinear relationship with motor unit activation at stimulation rates > 8 Hz (Orizio et al. 1999; Yoshitake and Moritani 1999; Bichler 2000). The MMG response also exhibits an association with oxygen uptake kinetics and therefore corroborates information related to muscle metabolism (Stout et al. 1997; McKay et al. 2009). This therefore places a premium on examining the fatigue‐based MMG response patterns in order to examine the contractile state of the muscle and infer upon adaptive patterns of motor control.

The first studies (Barry et al. 1985; Stokes and Dalton 1991) that examined the MMG response in the presence of fatigue reported that the time and frequency components of the signal (i.e., MMG amplitude and frequency) were influenced by the intensity and duration of muscle activity. The MMG amplitude and mean frequency (MNF) both provide information related to motor unit activation patterns (Orizio et al. 2003). MMG amplitude primarily reflects the number of active motor units and their firing rates, with larger motor unit spike amplitudes corresponding with greater MMG amplitude values (Orizio 1993; Yoshitake and Moritani 1999; Bichler 2000). Similarly, electrical stimulation studies (Barry and Cole 1990; Orizio et al. 1996; Yoshitake et al. 2002) have shown that the MMG MNF matches the stimulus frequency, and has therefore been hypothesized to reflect the unfused motor unit firing rates (Orizio et al. 1999, 2003; Beck et al. 2007a). Moreover, there is evidence that shows that MMG is influenced by training status and the relative muscle fiber composition (Orizio and Veicsteinas 1992; Beck et al. 2007a; Trevino et al. 2016). For instance, marked differences have been observed in the MMG response, with and without the presence of fatigue, for the vastus lateralis of long‐distance runners and sprinters (Orizio and Veicsteinas 1992) as well as aerobically trained versus resistance trained individuals (Beck et al. 2007b).

Fatigue has been defined as any exercise‐induced reduction in the ability of the muscle to generate force (Gandevia 2001). The task‐dependent nature of fatigue has encouraged the examination of the physiological processes specifically involved with task failure (Hunter et al. 2004; Madeleine and Farina 2008). Basmajian and De Luca (1985) recommended that fatigue should be recognized as a time‐dependent process instead of a specific point. This concept is evident during sustained force tasks, as the progression of fatigue will eventually result in the failure to maintain force despite a maximal effort (i.e., task failure). Previous investigations that have employed MMG to assess neuromuscular fatigue have used a variety of fatiguing protocols; some have predefined the time duration (Beck et al. 2007b) or the number of repetitions (Kouzaki et al. 1999; Perry‐Rana et al. 2002), and others have assessed the MMG‐force relationship in fatigued muscle (Orizio et al. 2003), yet very little is known about MMG and task failure (Madeleine and Farina 2008). Although boundary conditions exist, it is now recognized that the rate of fatigue development and the corresponding motor control adaptations are highly individualistic (Blain et al. 2016; Contessa et al. 2016). Moreover, the muscle force twitch properties in the presence of fatigue confer motor unit firing adaptations, and the magnitude of these adaptations are unique between individuals (Adam and De Luca 2005; Contessa et al. 2016; Herda et al. 2016). Recent observations show that task failure corresponds with a specific level of intramuscular metabolic perturbation during single‐joint and whole‐body exercise (Burnley et al. 2010; Hureau et al. 2014). Interestingly, it has been observed (Booghs et al. 2012) that motor control adjustments, not muscle oxygenation levels, are associated with failure times during sustained high‐intensity isometric force tasks. Investigations are therefore warranted (Zwarts and Keidel 1991; Ryan et al. 2007; Madeleine and Farina 2008; Cochrane‐Snyman et al. 2016) to examine individual fatigue‐based motor control adaptations in relation to failure times in order to form generalizable conclusions about rate‐limiting processes while still allowing for interindividual differences to be considered.

The ability to associate individual patterns of change in MMG with failure times would link fatigue‐based motor unit adaptations, notably contractile capacity, with endurance performance. This could be of use for clinicians when evaluating rehabilitation progression or developing exercise prescription measures. Therefore, the purpose of this study was to examine the individual MMG response during, and at the failure point of a sustained maximal and submaximal isometric force task. It was hypothesized that, due to the greater rate of fatigue development for the maximal task, MMG would more closely associate with failure times but would be reduced to a similar degree compared to the submaximal task.

## Methods

### Subjects

Twenty healthy men (mean ± SD: age 25 ± 4 years; height = 180.0 ± 5.5 cm; body mass, 85.1 ± 12.0 kg) volunteered to participate in this study. The study was approved by the Institutional Review Board for Human Subjects at the University of Oklahoma in accordance with the ethical standards laid down in the Belmont Report. All subjects completed a health history questionnaire and signed an informed consent prior to testing. The subjects varied in physical activity level from sedentary to extremely active. Before testing, the subjects were instructed to arrive at the laboratory prepared for strength testing (no heavy exercise in the past 48 h) and each visit was scheduled around the same time of day (±2 h).

### Isometric testing

**Figure 1:**
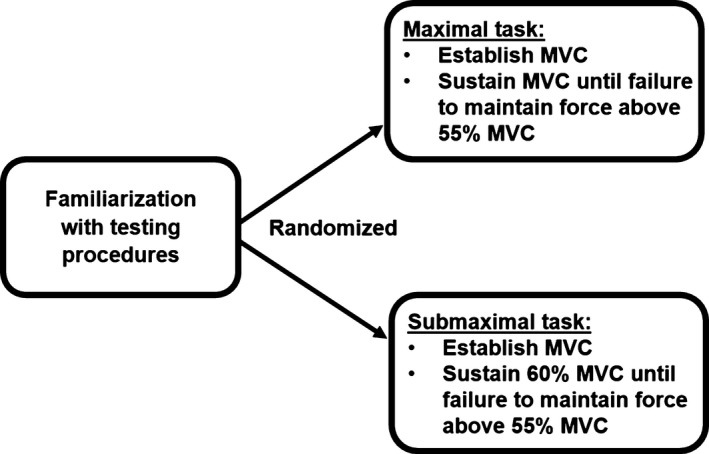
A diagram for the experimental design and procedures of the present study.

The subjects reported to the laboratory on three nonconsecutive occasions within a 2‐week period. The first visit was a simple familiarization with the equipment and testing procedures. The second and third visits required the subject to perform either a maximal or a submaximal isometric contraction of the dominant (based on throwing preference) elbow flexors until failure. Isometric force tasks were chosen for stronger MMG signal stationarity compared to dynamic contractions, and the biceps brachii muscle was selected due to its function as the chief elbow flexor (Basmajian and De Luca 1985). The subjects were seated in front of a custom built isometric strength testing apparatus so that the elbow rested on a pad, while their forearm was upright and their wrist within a cuff attached to a tension‐compression load cell (Model SSM‐AJ‐500, Interface, Inc., Scottsdale, AZ). A baseline maximal voluntary contraction (MVC_b_) was established by having the subjects perform three, 5 sec MVCs of the elbow flexors. Two minutes of rest were allowed between each trial. The highest force value from the three trials was designated as the MVC_b_. Then, the subjects sustained either a maximal or submaximal (60% MVC_b_) isometric contraction for as long as possible (Figure 1). Testing was terminated when force output could not be maintained above the same relative MVC fraction (55% MVC_b_). The subjects were unaware of the termination criteria, as well as the contraction time. The time to task failure was defined as the time‐course from the onset of the contraction to test termination. The subjects were provided with visual feedback from a force tracing template displayed on a monitor during the submaximal test. Prior to testing, the experimenter reminded each subject of the testing protocol, specifically addressing the need for maximal effort.

### MMG signal acquisition and processing

The surface MMG signals were detected from the biceps brachii with a miniature accelerometer (PCB Piezotronics, Model 352A24, bandwidth 1.0‐8000 Hz, dimensions: 0.19 x 0.48 x 0.28 in, mass 0.8 g, sensitivity 100 mV·g^−1)^. The accelerometer was positioned over the belly of the biceps brachii, and was secured to the skin with double‐sided foam tape. The skin was marked with permanent marker to ensure that the accelerometer placement could be replicated. The raw MMG signals were digitized at 2,000 samples/sec. The MMG signal was then high‐pass filtered (5 Hz) and low‐pass filtered (100 Hz) with a fourth‐order Butterworth filter. The MMG amplitude and MNF values were calculated in 1‐sec intervals during the length of the two fatigue tasks. The amplitude of the MMG signal was quantified as the root mean square (RMS). For the MMG MNF analyses, the MMG signal was filtered with a Hamming window and then processed with the discrete Fourier transform. The mean value and variance of the mean frequency and standard deviation of the power spectrum were calculated for each interval as described by Kwatny et al. (1970).

### Statistical analyses

The relationships between MMG amplitude and MMG MNF versus failure time were examined with polynomial regression for each individual. Using *X * =  time to failure, *Y * =  MMG amplitude or MMG MNF, and *a*_0_, *a*_1_, a_2_, and *a*_3_ = statistically determined regression coefficients as follows:

$$Y=a_{0}+a_{1}X\left(\text{linear model}\right)$$

$$Y=a_{0}+a_{1}X+a_{2}X^{2}\;\left(\text{quadratic model}\right)$$

$$Y=a_{0}+a_{1}X+a_{2}X^{2}+a_{3}X^{3}\left(\text{cubic model}\right)$$

Bivariate regression was used to determine the association between the rate of change for the MMG response and failure time within each composite model (i.e., group mean). The final MMG amplitude and MNF values were determined by averaging those values during the last 3 sec of the respective task. Paired samples *t* tests were used to compare the final MMG amplitude and MMG MNF values between the two tasks. The 95% CI for the mean difference for each paired samples *t* test was computed and Cohen's *d* was used to assess the effect size for the paired samples comparison (Cohen 1988). Cohen's *d* values of 0.2, 0.5, and 0.8 are used to characterize small, medium, and large effect sizes, respectively. An alpha level of 0.05 was used to determine statistical significance.

## Results

**Figure 2:**
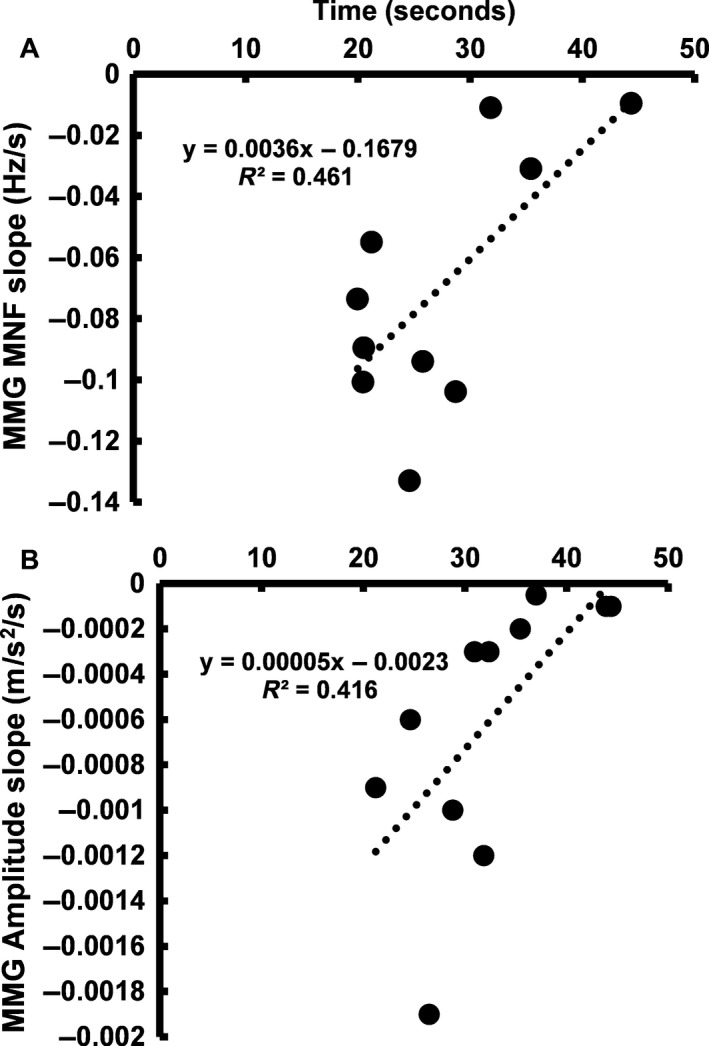
The relationship between failure time and the rate of change for MMG MNF (A: *n* = 10, *P* = 0.03) and MMG amplitude (B: *n* = 11, *P* = 0.03) for the composite models during the maximal task.

**Figure 3:**
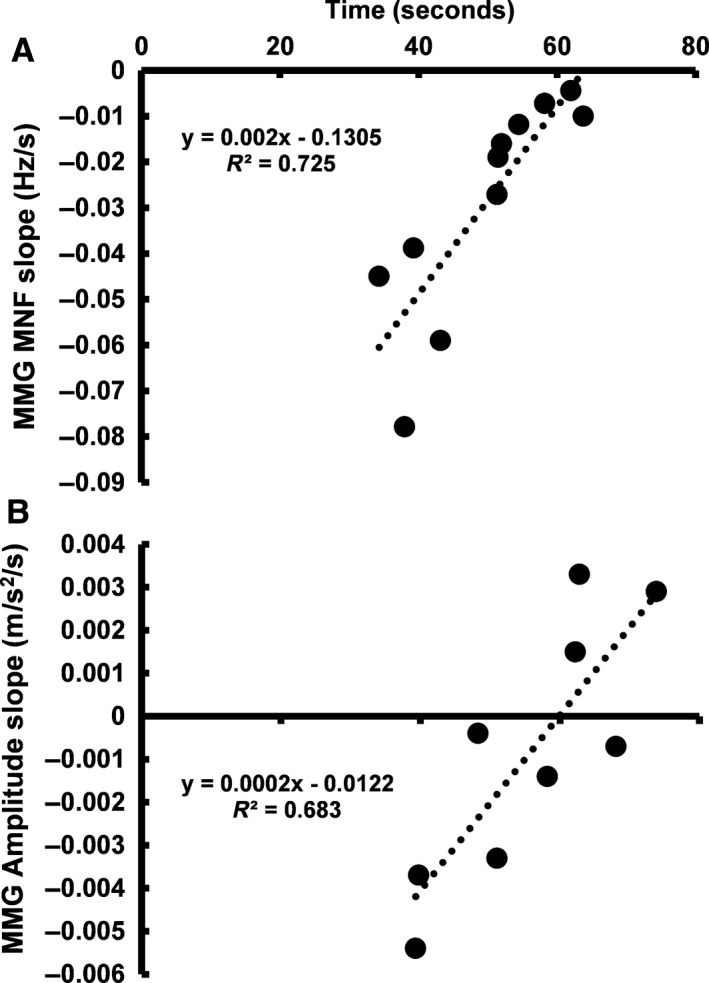
The relationship between failure time and the rate of change for MMG MNF (A: *n* = 11, *P* < 0.01) and MMG amplitude (B: *n* = 9, *P* < 0.01) for the composite models during the submaximal task.

**Figure 4:**
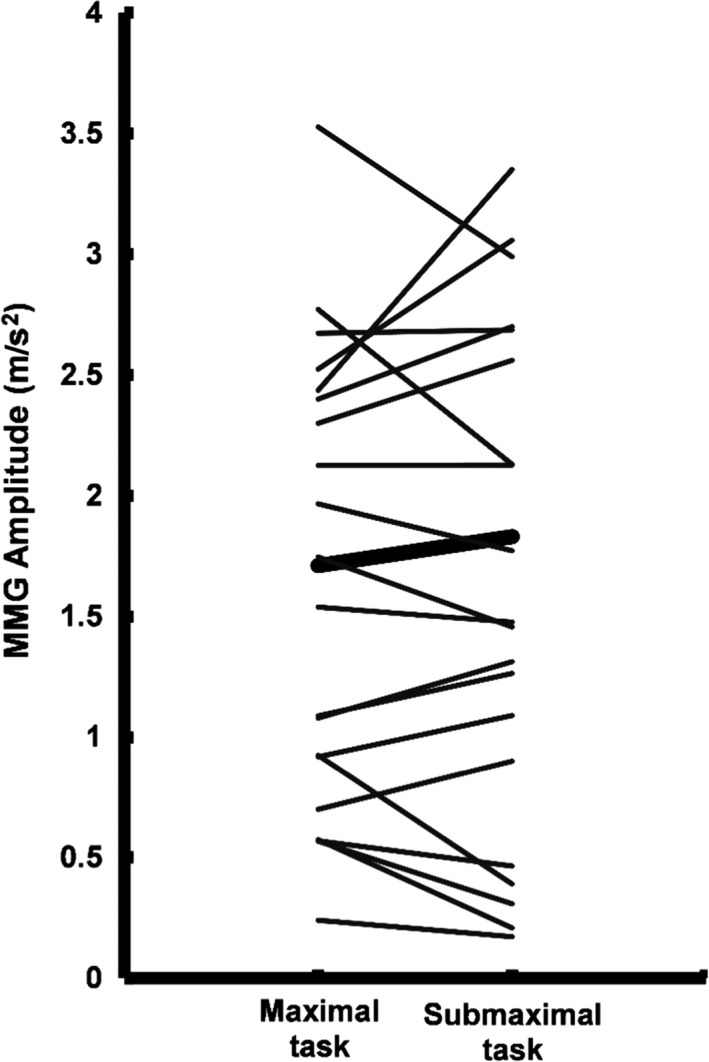
The final MMG amplitude values for the maximal and submaximal fatigue tasks are shown for each individual. The thick bar represents the group mean (*n* = 20, *P* = 0.741).

**Figure 5:**
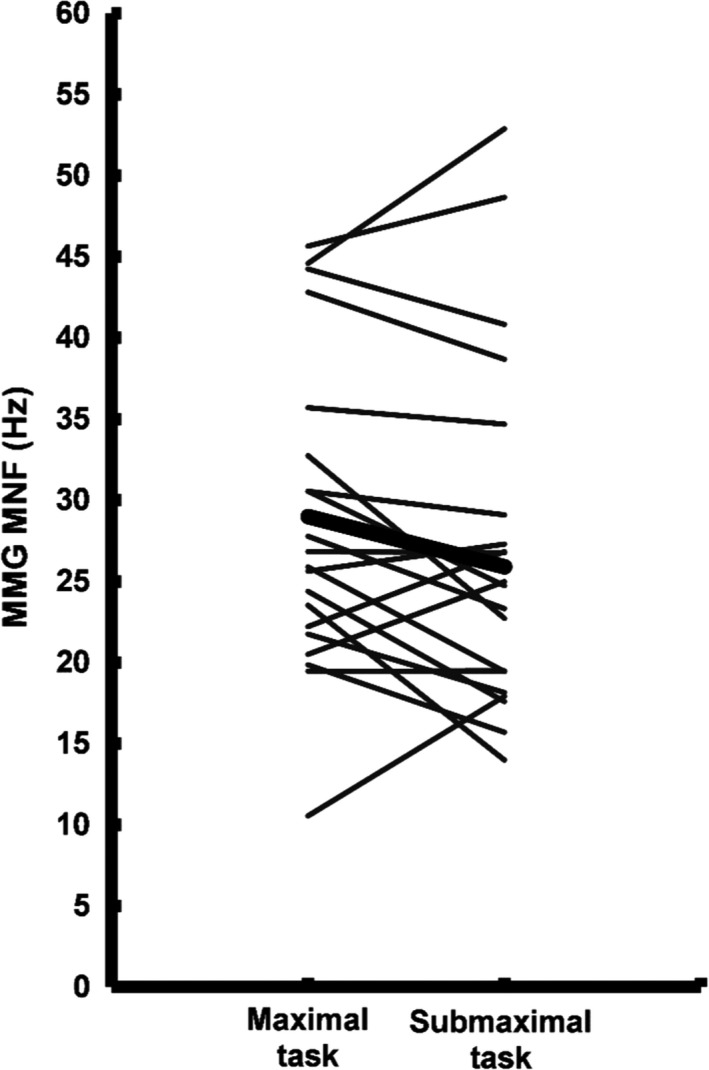
The final MMG MNF values for the maximal and submaximal fatigue tasks are shown for each individual. The thick bar represents the group mean (*n* = 20, *P* = 0.108).

The results of the polynomial regression analysis for the relationships between MMG amplitude and MNF versus time to failure for each individual are presented in Table 1. The response patterns for MMG amplitude and MMG MNF during the maximal task were best fit with cubic and quadratic models, respectively. The composite MMG amplitude and MNF response patterns were both best fit with quadratic models during the submaximal task. Bivariate regression showed that the rate of change within the composite models for the MMG MNF (*R* ^2^ = 0.461, *n* = 10, *P* = 0.031; Fig. 2A) and amplitude (*R* ^2^ = 0.416, *n* = 11, *P* = 0.032; Fig. 2B) responses during the maximal task were associated with failure times. Similarly, the rate of change for MNF (*R* ^2^ = 0.725, *n* = 11, *P* < 0.01; Fig. 3A) and MMG amplitude (*R* ^2^ = 0.683, *n* = 9, *P* < 0.01; Fig. 3B) during the submaximal task were also associated with failure times. The results from the paired samples *t* test indicated that there were no significant mean differences in the final MMG amplitude (maximal versus submaximal: 1.71 ± 1.25 vs. 1.83 ± 1.93 m·s^−2^, 95% CI: −0.930, 0.674, Cohen's *d *=* *0.07, *P* = 0.741) and MMG MNF (maximal vs. submaximal: 29.0 ± 10.5 vs. 25.9 ± 11.7 Hz, 95% CI: −0.750, 6.99, Cohen's *d *=* *0.278, *P* = 0.108) values between the two tasks. Figures 4 and 5 display the individual and mean data for the final MMG amplitude and MMG MNF values for both tasks.

**Table 1:** The individual polynomial regression models for the MMG amplitude and MMG MNF versus time to failure relationships for the maximal and submaximal tasks

Subject	MMG Amplitude				MMG MNF			
	Maximal task		Submaximal task		Maximal task		Submaximal task	
	Model	*R* ^2^	Model	*R* ^2^	Model	*R* ^2^	Model	*R* ^2^
1	Cubic	0.86	NS	NS	Quadratic	0.44	Linear	0.70
2	Linear	0.55	Cubic	0.57	Quadratic	0.42	Quadratic	0.72
3	Cubic	0.33	Cubic	0.49	Quadratic	0.22	Quadratic	0.18
4	Cubic	0.55	NS	NS	Quadratic	0.45	Quadratic	0.45
5	Cubic	0.50	Cubic	0.79	Quadratic	0.39	Quadratic	0.39
6	Cubic	0.29	Linear	0.11	Quadratic	0.19	Cubic	0.40
7	Linear	0.47	NS	NS	Linear	0.34	Cubic	0.30
8	NS	NS	Quadratic	0.59	Quadratic	0.44	Linear	0.86
9	NS	NS	Quadratic	0.49	Quadratic	0.39	Quadratic	0.78
10	Quadratic	0.70	Quadratic	0.29	NS	NS	Cubic	0.88
11	Cubic	0.72	Quadratic	0.78	Quadratic	0.26	Quadratic	0.75
12	Quadratic	0.55	Quadratic	0.46	Quadratic	0.73	Linear	0.82
13	Cubic	0.54	Cubic	0.37	Quadratic	0.47	Cubic	0.84
14	Quadratic	0.48	Cubic	0.37	NS	NS	Quadratic	0.80
15	Quadratic	0.81	Linear	0.75	Linear	0.41	NS	NS
16	Linear	0.75	Cubic	0.86	NS	NS	Quadratic	0.17
17	Cubic	0.87	Quadratic	0.85	Cubic	0.64	Quadratic	0.67
18	Cubic	0.86	Quadratic	0.58	Cubic	0.63	Linear	0.52
19	Quadratic	0.39	Cubic	0.79	Cubic	0.52	Cubic	0.82
20	Cubic	0.85	Quadratic	0.34	Cubic	0.66	Quadratic	0.68
Composite	Cubic	0.62	Quadratic	0.56	Quadratic	0.45	Quadratic	0.62

Regression coefficients that were not significantly different from zero are denoted NS.

## Discussion

This study employed MMG to examine the time‐dependent process of muscle fatigue during a maximal and submaximal isometric force task sustained to failure. The findings of this study show that the MMG response for the biceps brachii closely monitored the fatigue‐based changes in muscle function that progressed to task failure. Moreover, the results demonstrated that the rate of change for MMG amplitude and MNF were associated with failure time for both tasks. These results also suggest contractile capacity was reduced to a similar extent at the failure point between the two tasks. The composite models show that the majority of subjects exhibited nonlinear MMG response patterns for both the maximal and submaximal tasks. These findings are similar to others that have observed variable, curvilinear MMG response patterns when examining MMG‐fatigue relationships (Orizio et al. 1989; Orizio and Veicsteinas 1992; Perry‐Rana et al. 2002; Cochrane‐Snyman et al. 2016). These observations provide support for the notions that fatigue‐based changes in motor unit activity are reflected in the MMG response, are highly variable between individuals, and contribute to task failure.

The time‐dependent nature of neuromuscular fatigue has encouraged investigations to characterize the physiological processes specifically involved with task failure (Hunter et al. 2004; Madeleine and Farina 2008). This novel approach can distinguish unique motor control strategies between different tasks and may therefore provide valuable information regarding the different rate‐limiting patterns that are associated with task failure. The majority of studies that have used MMG as a tool to describe fatigue‐based changes in neuromuscular function have typically employed sustained exercise to exhaustion (Orizio et al. 1989; Orizio and Veicsteinas 1992; Housh et al. 2000), and some have predefined the duration (Beck et al. 2007b; Cochrane‐Snyman et al. 2016) or number of repetitions (Kouzaki et al. 1999; Perry‐Rana et al. 2002) to be completed, yet only Madeleine and Farina (2008) have combined MMG and task failure. A unique aspect of this study was that the subjects were required to sustain a maximal and submaximal force to the same predetermined failure point of their relative force loss. Comprehensive reviews (Orizio et al. 2003; Beck et al. 2007a; Islam et al. 2013) have shown that during sustained, fatiguing isometric muscle actions, the relative shifts in the MMG components strongly reflect fatigue‐based changes in motor unit behavior. The MMG response during sustained isometric muscle actions provides support for this, as the MMG amplitude has generally been shown to increase, remain stable, or decrease across time as the relative intensity is increased (Orizio et al. 1989). Generally speaking, the increase in MMG amplitude across time has been attributed to the recruitment of larger, higher‐threshold motor units, while the curvilinear decline in MMG amplitude that has been observed at higher intensities is believed to reflect decreased high‐threshold motor unit activity, either through their decruitment, the fusion of their force twitches, or increased firing rates of the unfused motor units (Orizio 1993). Similarly, fatigue reduces the MMG MNF, and during sustained muscle actions, a nonlinear shift toward lower frequencies has been observed (Orizio et al. 1989; Beck et al. 2007a). The MMG MNF shift is indicative of fatigue‐based alterations in motor unit firing rates (Orizio et al. 2003; Beck et al. 2007b). There are several physiological conditions that may contribute to the reduction in the MMG MNF in the presence of fatigue, though the dominant factor is likely related to a prolonged motor unit twitch time (Orizio et al. 1999). These nonlinear response patterns were observed in this study and emphasize the fundamental interaction between motor unit firing properties and the responsiveness of the muscle force twitch during sustained force tasks (Adam and De Luca 2005; Contessa et al. 2016).

This study observed that at task failure, there was a comparable level of contractile disturbance between the maximal and submaximal tasks. MMG amplitude and MNF values were not significantly different at the failure point of the two tasks, and displayed similar relative changes from their maximum values. More specifically, MMG amplitude was decreased by 50% and 47%, and MMG MNF fell by 34% and 40% during the maximal and submaximal task, respectively. The observations for MMG amplitude suggest that there were individually specific levels of tetanic fusion for the muscle fibers of the active motor units, and although necessarily speculative, the MMG MNF values at failure may reflect comparable adaptations in motor unit firing rates conferred by prolonged force twitch kinetics. Figures 4 and 5 illustrate the individual along with the mean values at failure for MMG amplitude and MNF, respectively. The MMG is mechanical in nature and provides information regarding the contractile properties of the summed motor unit twitches (Orizio et al. 1999; Yoshitake and Moritani 1999). Specifically, the MMG response is closely related with twitch duration and half relaxation times (Yoshitake et al. 2002), as well as whole muscle relaxation with or without the presence of fatigue (Cè et al. 2017). Previous data (Orizio et al. 1999; Bichler and Celichowski 2001; Yoshitake et al. 2002) have shown that the magnitude of the motor unit force twitch, as well as their fatigue‐based elongation properties, are reflected in the rate of change in the MMG response and are heavily influenced by tetanic fusion. Given the MMG values at the failure point, it is possible that the degree of impairment in force twitch mechanics were similar at task failure, though this is speculative as this study did not directly measure evoked twitch responses or intramuscular metabolite concentrations. In addition, the curvilinear response patterns observed in this study may be explained by unique fatigue‐based motor control strategies that result from different expressions of the myosin heavy chain isoform within the contributing motor units (Contessa et al. 2016; Herda et al. 2016; Trevino et al. 2016). This explanation is supported by studies that have observed smaller absolute values and patterns of change for MMG amplitude and MNF for individuals with larger proportions of the type 1 myosin heavy chain isoform (Orizio and Veicsteinas 1992; Beck et al. 2007b; Trevino et al. 2016).

In this study, MMG amplitude displayed cubic and quadratic decreases during the maximal and submaximal task. It is important to consider that the rate and magnitude of the decrease for MMG are the result of similar fatigue‐based mechanisms. During the maximal task, there was a general trend for MMG amplitude to exponentially decline, modestly increase, and then further decrease until task failure. The transient increase at approximately half of contraction time is similar to previous observations (Bichler and Celichowski 2001) and likely reflects twitch potentiation (Adam and De Luca 2005; Miller et al. 2017). The exponential drop for MMG amplitude is also consistent with previous reports (Orizio and Veicsteinas 1992; Orizio et al. 1999) and demonstrates reduced motor unit activity, most notable is likely the simultaneous fusion of force twitches and high‐threshold motor unit decruitment. The submaximal MMG amplitude response was more variable and showed two forms of quadratic behavior, either steadily increasing until roughly half of contraction time and then decreasing, or increasing exponentially near task end after maintaining relatively constant levels. Beck et al. (2007b) observed similar, disparate MMG amplitude patterns for the vastus lateralis of aerobic and resistance trained individuals during a sustained 50% MVC of the knee extensors. Specifically, MMG amplitude exhibited quadratic response patterns for the resistance trained group, whereas the aerobically trained group displayed a linear increase across time. The MMG MNF response showed a quadratic decline for both tasks and was generally more uniform compared to MMG amplitude. This curvilinear reduction is consistent with previous reports for fatiguing isometric contractions (Orizio and Veicsteinas 1992; Kouzaki et al. 1999; Madeleine and Farina 2008) and has been linked with a fatigue‐induced deterioration in force twitch mechanics along with corresponding alterations in motor unit firing rates (Orizio et al. 2003; Beck et al. 2007a). Orizio and Veicsteinas (1992) found that during a sustained maximal contraction of the knee extensors, the MMG MNF response for the vastus lateralis demonstrated a greater rate of change for sprinters compared to long distance runners. The sprinters also had a shorter time to exhaustion, and interestingly, maintained greater absolute MMG MNF values at exhaustion compared to the long distance runners. Madeleine and Farina (2008) used an MMG grid to examine task failure during a sustained contraction of the trapezius muscle at 20% MVC. The authors reported that failure time was positively associated (*R* ^2^ = 0.36) with the ratio of MMG amplitude at task failure compared to MVC values (i.e., activation ratio). In other words, this association implies that subjects that had longer endurance times recruited a greater relative fraction of their motor units. These studies (Orizio and Veicsteinas 1992; Beck et al. 2007b; Madeleine and Farina 2008) provide a basis for the interpretation of the current findings. The negative associations between the rates of change for MMG and failure times within the present experimental design is likely related to the unique combination of myosin heavy chain isoform expression (Orizio and Veicsteinas 1992; Beck et al. 2007b; Trevino et al. 2016) and the corresponding motor control adaptations between individuals (Madeleine and Farina 2008; Contessa et al. 2016). That is, together with the individually specific levels of MMG at failure, the greater rate of change for MMG amplitude and MNF for individuals with shorter failure times suggests the time‐dependent impairments in the properties of the contributing motor units were a rate‐limiting factor that led to force failure.

Many studies (Housh et al. 2000; Perry‐Rana et al. 2002; Cochrane‐Snyman et al. 2016; Cè et al. 2017) have suggested that the MMG response during fatiguing exercise may support the muscle wisdom hypothesis (Marsden et al. 1983), a theory that postulates a sensory‐mediated reduction in the firing rates of a motor unit to match the fatigue‐induced elongation in its twitch mechanics, sparing metabolic cost by utilizing the force‐frequency relationship. However, there has been considerable discussion on the generalizability of such a mechanism (Garland and Gossen 2002; Fuglevand and Keen 2003). Another tenable theory ‘the sensory tolerance limit’ has been recently developed and links peripheral and central factors of fatigue into a summative feedback model that incorporates the magnitude of sensory input and motor output with task failure (Hureau et al. 2016). There is compelling data (Burnley et al. 2010; Vanhatalo et al. 2010; Amann et al. 2013; Hureau et al. 2014; Blain et al. 2016) that supports this hypothesis which has shown that voluntary termination of single‐joint and whole‐body exercise corresponds with an individually specific level of intramuscular metabolic perturbation. Elevated concentrations of intramuscular metabolites exacerbate the development of fatigue by impairing crossbridge performance (Allen and Trajanovska 2012) and stimulating group III/IV muscle afferents (Gandevia 2001). The inhibitory feedback delivered to the central nervous system by these muscle afferents has been linked with reduced central motor drive and increased perceived exertion (Amann et al. 2013; Blain et al. 2016; Broxterman et al. 2017). Studies that employed muscle afferent blockade have shown that group III/IV muscle afferents play a critical role in maintaining a relative level of muscle homeostasis during isometric and whole‐body exercise and have therefore been corroborated as an integral part of the feedback loop in the sensory tolerance limit hypothesis (Burnley et al. 2010; Amann et al. 2013; Hureau et al. 2014; Blain et al. 2016; Broxterman et al. 2017). Similar evidence for unique sensory tolerance between individuals was demonstrated by Cochrane‐Snyman et al. (2016), who observed large degrees of inter‐individual variability for the patterns of MMG response when subjects performed cycling exercise at a constant level of perceived exertion. In this context, it is interesting to consider the individualistic rate of change in force twitch kinetics together with the stimulation of group III/IV muscle afferents in regards to failure times during fatiguing exercise. Collectively, the associations observed in this study between the rates of change for MMG and failure times lends support for the existence of a peripheral sensory‐mediated reflex that corresponds with task failure.

In summary, by examining the MMG response and the time‐dependent process of neuromuscular fatigue, it was observed that the time and frequency components of the MMG signal were associated with failure times during sustained, high‐intensity isometric contractions. These associations suggest that the fatigue‐induced diminution in force twitch kinetics were a rate‐limiting process that contributed to task failure. These data also demonstrate that there were comparable levels of peripheral disturbance between the two tasks, suggesting that task failure coincided with similar reductions in the contractile capacity of the biceps brachii. The variable MMG responses indicate different patterns of mechanical work and it is possible that unique muscle fiber type expressions and corresponding motor control adaptations between individuals could account for these discrepancies, though motor unit firings and myosin heavy chain isoform expressions were not directly measured in this study. The interpretation of these findings are therefore limited solely to MMG. Future studies that link MMG responses with intramuscular metabolite concentrations in the presence of fatigue, along with direct measurements of motor unit firing patterns during voluntary contractions will provide a deeper understanding of the compensatory interaction between motor unit firing behaviors and the properties of the muscle force twitch.

## Conflict of Interest

None declared.
